# Maturity-onset diabetes of the young secondary to HNF1B variants (HNF1B-MODY): a series of 10 patients from a single diabetes center

**DOI:** 10.1186/s13098-022-00964-0

**Published:** 2023-02-15

**Authors:** Sara Amaral, Ana Palha, Paula Bogalho, José Silva-Nunes

**Affiliations:** 1 Department of Endocrinology, Diabetes and Metabolism, Centro Hospitalar Universitário Lisboa Central, Lisbon, Portugal; 2 NOVA Medical School/Faculdade de Ciencias Medicas, Universidade Nova de Lisboa, Lisbon, Portugal; 3 Health and Technology Research Center (H&TRC), Escola Superior de Tecnologia da Saude de Lisboa, Lisbon, Portugal

**Keywords:** Diabetes, Maturity-onset diabetes mellitus, MODY, HNF1B, Chronic kidney disease

## Abstract

**Background:**

Maturity-Onset Diabetes of the Young (MODY) is an autosomal dominant condition and represents 1–5% of all cases of diabetes mellitus. MODY is often misdiagnosed as type 1 or type 2 diabetes. The rare subtype 5 (HNF1B-MODY) is due to hepatocyte nuclear factor 1β (*HNF1B*) molecular alteration and is remarkable for its multisystemic phenotypes characterized by a broad spectrum of pancreatic and extra-pancreatic clinical manifestations.

**Methods:**

Retrospective study of patients with HNF1B-MODY diagnosis followed in the Centro Hospitalar Universitário Lisboa Central (Lisbon, Portugal). Demographic data, medical history, clinical and laboratory data, follow-up and treatment procedures were obtained from electronic medical records.

**Results:**

We found 10 patients with *HNF1B* variants (7 index cases). The median age at diabetes diagnosis was 28 (IQR 24) years and the median age at HNF1B-MODY diagnosis was 40.5 (IQR 23) years. Six patients were initially misclassified as type 1 and 4 as type 2 diabetes. The average time between diabetes diagnosis and the diagnosis of HNF1B-MODY was 16.5 years. Diabetes was the first manifestation in half of the cases. The other half presented with kidney malformations and chronic kidney disease at pediatric age as the first manifestation. All these patients were submitted to kidney transplantation. Long-term diabetes complications included retinopathy (4/10), peripheral neuropathy (2/10) and ischemic cardiomyopathy (1/10). Other extra-pancreatic manifestations included liver test alterations (4/10) and congenital malformation of the female reproductive tract (1/6). History of a first-degree relative with diabetes and/or nephropathy diagnosed at a young age was present in 5 of the 7 index cases.

**Conclusions:**

Despite being a rare disease, HNF1B-MODY is underdiagnosed and often misclassified. It should be suspected in patients with diabetes and CKD, especially when diabetes appears at a young age, a family history is present, and nephropathy appears before/shortly after the diagnosis of diabetes. Presence of unexplained liver disease increases the degree of suspicion for HNF1B-MODY. Early diagnosis is important to minimize complications and to allow familial screening and pre-conception genetic counseling.

*Trial registration* not applicable due to the retrospective nature of the study, non-interventional.

## Introduction

Maturity-Onset Diabetes of The Young (MODY) is the most common type of monogenic diabetes. It is a heterogeneous group of diseases, with autosomal dominant inheritance, caused by gene variants that result in pancreatic beta-cell dysfunction [1]. Although MODY accounts for only 1–5% of all diabetes cases it has significant implications [2]. There are now at least 14 different known MODY subtypes corresponding to pathogenic variants in the same number of genes (*HNF4α, GCK, HNF1α, PDX1, HNF1B, NEURO1, KLF11, CEL, PAX4, INS, BLK, ABCC8, KCNJ11* and *APPL1*). The different MODY subtypes vary with respect to age of onset, response to treatment and the presence of extra-pancreatic manifestations [3].

The subtype 5 (HNF1B-MODY) is a rare form of the disease caused by *HNF1B* gene variants or whole gene deletions [4] and it accounts for 2–5% of all MODY subtypes [5]. De novo genetic variants account for 50–60% of all reported cases [5].

HNF1B is a transcription factor of the homeodomain-containing transcription factor superfamily and is found in a wide range of tissues such as liver, intestine, stomach, lung, and pancreas. It is expressed in the early stage of embryonic development, being vital for the development of the nephron and the pancreas [6]. *HNF1B* contains 9 exons and is located on chromosome 17q12 [7]. Heterozygous genetic variations comprise base substitutions leading to missense, nonsense, small deletions or insertions, frameshift and splicing mutations; in some cases, complete gene deletions have been described. Most are localized in the first four exons of the gene, with exons 2, 4, and the intron 2 splice site being hotspots. In 50% of cases, HNF1B-MODY results from a 17q12 deletion encompassing 15 genes, including *HNF1B* [8, 9].

The *HNF1B* gene plays a critical role on the transcriptional factors network that manage the process of differentiation of pancreatic multipotent cells to endocrine, ductal and acinar cells [10]. *HNF1B* defects could lead to distinct alterations in pancreas morphology ranging from complete or partial pancreatic agenesis to diffuse pancreatic atrophy [11]. Pancreatic exocrine deficit has been described with a prevalence of 20–75%. It is usually asymptomatic and can be documented by reduced fecal elastase [12, 13, 18]. Diabetes results from a combination of β-cell dysfunction and insulin resistance. Typically, the onset of diabetes is during early life, with a mean age of presentation of around 25 years of age [14]. Due to the progressive nature of β-cell dysfunction and to renal failure, most affected individuals progressively require insulin. The development of diabetes-related complications depends on its evolution time [15].

*HNF1B* also plays an important role in the growth of collecting ducts, renal pelvis and ureter, and differentiation of the metanephric mesenchyme, which are all key elements for the development of the nephron and collecting system. Variants in the *HNF1B* gene are the most frequent cause of monogenic congenital anomalies of the kidney and urinary tract and remain one of the major causes of chronic kidney disease (CKD) in the prenatal and childhood period [7, 16]. Liver dysfunction is also prevalent in HNF1B-MODY patients. Histological studies show bile ductopenia, steatosis, and periportal fibrosis, which could lead to cases of neonatal or adult cholestatic hepatopathy [7]. Congenital malformations of the female reproductive tract are also described in these patients and include bicornuate uterus, uterus didelphys, rudimentary uterus and vaginal atresia [14]. In addition, hyperuricemia with gout of early onset, hypokalemia and hypomagnesemia can also be present. At least, intellectual disability has been reported in up to 10% of patients.

HNF1B-MODY express variable multisystemic phenotypes with a wide spectrum of pancreatic and extra-pancreatic clinical manifestations, ranging from isolated diabetes or kidney disease to multiorgan disorders [13].

Due to HNF1B-MODY variable multisystemic phenotypes, in this study the authors aim to describe and characterize the different clinical presentations of this disease.

## Methods

The authors conducted an observational study, descriptive and retrospective. Written informed consent was obtained from all individuals involved after full explanation of the purpose and nature of the study. The study was approved by the Ethics Committee of the Centro Hospitalar Universitário Lisboa Central (CA 350/2022).

## Study population

The study sample consisted of 10 patients with HNF1B molecular defects followed from January 2013 to December 2021 in the Department of Endocrinology, Diabetes and Metabolism—Centro Hospitalar Universitário Lisboa Central, in Lisbon. Demographic data, medical history, clinical and laboratory data, follow-up and treatment procedures were obtained from electronic medical records. Data are shown as medians and inter-quartile ranges (IQRs) or as numbers and percentages.

## Genetic analyses

Blood was collected and DNA extracted. Promoter region, coding sequences and adjacent intronic regions of GCK (NM_000162.3), HNF1A (NM_000545.6), HNF1B (NM_000458.3) and HNF4A (NM_000457.4) were amplified and Sanger sequenced employing a cascade screening strategy. Large rearrangements studies were conducted with MLPA. In silico analysis was performed and variants classified according to the American College of Medical Genetics (ACGM) recommendations.

## Results

The authors found 10 patients (6 females and 4 males) with *HNF1B* molecular defects (7 index cases and 3 relatives). The median follow-up time was 6 years. The median age at HNF1B-MODY diagnosis was 40.5 (IQR 23) years. Clinical and laboratory data are summarized in Table 1. At last follow-up, median age of the patients was 46.5 (IQR 18) years.

**Table 1:** Summary of clinical and laboratory data

Patient	1	2	3	4	5	6	7	8	9	10
Case	Index	Familial	Index	Familial	Index	Index	Index	Index	Familial	Index
Sex	Female	Male	Female	Female	Female	Female	Male	Male	Female	Male
Genotype	c.827G > Ap.(Arg276Gln)	c.827G > Ap.(Arg276Gln)	c.1-?_1674 + ? del	c.1-?_1674 + ? del	c.1147C > T p.(Gln383*)	c.988C > Tp.(Leu330Phe)	c.494G > Ap.(Arg165His)	c.526C > Tp.(Gln176*)	c.526C > Tp.(Gln176*)	c.493C > Tp.(Arg165Cys)
BMI (kg/m^2^)^a^	26.4	25.3	22.0	24.5	26.7	19.5	20,4	31.0	28.4	27.1
Current age (y/o)	49	46	41	72	30	59	29	41	61	47
Age at MODY diagnosis (y/o)	42	39	35	69	29	58	28	38	60	42
Age at diabetes diagnosis (y/o)	28	28	21	42	29	49	9	13	40	16
Diabetes evolution time (y)	21	18	20	30	1	10	20	28	21	31
Current diabetestreatment	Insulin	Insulin	Insulin	Insulin	Oral agents	Insulin	Insulin	Insulin	Insulin	Insulin
Complications (renal not included)	DR^b^	None	DR	None	None	None	ICM^c^	DR, DN^d^	None	DR, ICM
C-peptide	0.5	0.2	0.5	0.4	2.8	3.8	0.9	0.2	0.3	0.1
HbA1c (%)	6.3	6.5	7.8	7.8	6.2	7.1	9.7	9.5	8.9	9.3
Renal malformation	URH^g^	SK^h^	URH	No	Unknown	No	BRH^i^	No	No	URH
Transplantation	Kidney	Kidney Liver	No	No	Kidney	No	No	Kidney Pancreas	No	Kidney Pancreas
CKD	Yes	Yes	Yes	No	Yes	No	Yes	Yes	Yes	Yes
CKD prior to diabetes (years)	13	20	10	–	16	–	1	–	–	–
CKD after diabetes (years)	–	–	–	–	–	–	–	2	19	5
eGFR^f^	77	55	HD	52	76	99	17	51	30	17
Albumin urinary excretion	Y	Y	N/A	N	N	N	N/A	Y	N	Y
Hypertension	Y	Y	Y	Y	N	Y	Y	Y	Y	Y
Elevated liver enzymes	Y	Y	N	N	N	N	N	N	Y	Y
Hypomagnesemia	N	N	N	N	Y	N	N	N	N	N
Hyperuricemia	Y	Y	N	N	Y	N	N	N	Y	Y
Reproductive tract malformation	N	N/A	N	N	Y	N	N/A	N/A	N	N/A

^a^*BMI body mass index was calculated based on* anthropometric measurements from the last observation, ^b^*DR* diabetic retinopathy, ^c^*ICM* ischemic cardiomyopathy, ^d^diabetic neuropathy, ^e^C-peptide plasma concentration: reference range 0.9–7.1 ng/mL, ^f^*eGFR* estimated glomerular filtration rate (ml/min/1.73 m2), ^g^*URH* unilateral renal hypoplasia, ^h^*SK* solitary kidney, ^i^*BRH* bilateral renal hypoplasia

The average time between diagnosis of diabetes and HNF1B-MODY diagnosis was 16.5 years. The median age at diabetes diagnosis was 28 (IQR 24) years. Six patients were initially misclassified as type 1 diabetes and four patients as type 2 diabetes. The clinical presentation at diabetes onset was variable. Most patients (6/10) reported polyuria and/or weight loss. Ketoacidosis occurred in 2 patients (patients 7 and 8). At diabetes onset, 6 patients began treatment with insulin. During follow-up, 3 more patients started insulin therapy; both patients 1 and 9 started insulin therapy 5 years after diabetes diagnosis and patient 4 started insulin therapy 25 years after diagnosis. Patient 5 was treated only with oral hypoglycemic agents (metformin 2 g/day and gliclazide 30 mg/day). The three related patients were reclassified as HNF1B-MODY after familial screening and had a previous diagnosis of type 2 diabetes. The average diabetes duration was 20 years. Long-term diabetes complications were present in 5 patients (5/10)—retinopathy (4/10), peripheral neuropathy (2/10) and ischemic cardiomyopathy (1/10). At last follow-up, islet cell (ICA), glutamic acid decarboxylase (GAD) and insulin-associated (IA-2) autoantibodies were negative in all patients except for patient 7 with anti-GAD 1,4 U/mL. Mean C-peptide plasma concentration at fasting was 0.48 ng/mL (reference range = 0.9–7.1 ng/mL). C-peptide plasma concentration at the time of diabetes diagnosis was not available. Median HbA1c on the last appointment was 7.8% (61.7 mmol/mol).

CKD was present in 8 patients. The diagnosis of CKD preceded the diagnosis of diabetes in 5 patients (on average, 12 years before). These patients were diagnosed in pediatric age and 4 of them had renal malformations: unilateral kidney hypoplasia (2/4), bilateral kidney hypoplasia (1/4) and solitary kidney (1/4). Five patients were submitted to kidney transplantation (kidney alone (2/5), combined kidney and pancreas (2/5), and combined kidney and liver (1/5); currently, all have a functioning kidney graft. At the time of transplantation, the median recipient age was 31 years (IQR 13.5). The average kidney graft survival was 10.2 years. Both patients submitted to pancreatic transplantation lost their graft. In patient 8, post-operative vascular complications (arterial thrombosis) occurred, resulting in complete pancreatic graft failure three days after transplantation. Patient 10 decreased insulin requirements in the first 14 days after transplantation (total daily dosage between 10 and 14 UI/day with plasma C-peptide at fasting ranging from 2.1 to 20 ng/mL), but the endogenous insulin secretion was never restored. During the transplant surgery an incidental discovery of an appendiceal well-differentiated neuroendocrine tumor (pT3 Nx Mx) was discovered and lymph node, peritoneal and colic metastasis developed during the follow-up. Regarding the five patients not submitted to kidney transplantation, the median eGFR at last observation was 30 mL/min/1.73m^2^ (IQR 63) and no albuminuria was observed (3/3). One patient (patient 3) is on hemodialysis awaiting kidney transplant surgery.

The other main extra-pancreatic manifestations (elevated liver enzymes, hypomagnesemia and hyperuricemia) were sought in all patients. The liver enzymes were elevated in 4 patients, hypomagnesemia was found in 1 patient and hyperuricemia in 5 patients. No patient has a history of gout crisis. All women had transvaginal ultrasound and a reproductive tract malformation (bicornuate uterus) was found 1 patient (1/6). Hypertension on last follow-up was present in 9/10 patients. During follow-up all the patients had at least one abdominal CT and no pancreatic malformations were reported. Pancreas exocrine functional test was performed in one patient without evidence of dysfunction. No patient had a complete neurocognitive assessment during follow-up. Antidepressant drugs were prescribed in 7/10 patients, anxiolytic in 5/10 patients and antipsychotic in 3/10 patients.

History of a first-degree relative with diabetes and/or nephropathy diagnosed at a young age (before 40 years-old) was present in 5 of the 7 index cases.

Table 2 describes genotypes and phenotypes. Patients 1 and 2 are siblings; patients 3 and 4 are daughter and mother, respectively; and patients 7 and 8 are son and mother, respectively.

**Table 2:** Genotype and phenotype of HNF1B-MODY patients

Patient	HNF1B variant	Type	ACMG classification	Novel/Known	Phenotype
1	c.827G > A p.(Arg276Gln)	*Missense*	Pathogenic	Known	Diabetes, CKD (kidney tx^a^), cholestatic liver disease
2	c.827G > A p.(Arg276Gln)	*Missense*	Pathogenic	Known	Diabetes, CKD (kidney and liver tx), cholestatic liver disease
3	c.1-?_1674 + ?del	Deletion	Pathogenic	Known	Diabetes, CKD, cholestatic liver disease
4	c.1-?_1674 + ?del	Deletion	Pathogenic	Known	Diabetes
5	c.1147C > T (p.Gln383*)	*Nonsense*	Likely pathogenic	Novel	Diabetes, CKD (kidney tx), uterine malformation
6	c.988C > T p. (Leu330Phe)	*Missense*	Uncertain significance	Novel	Diabetes
7	c.494G > A (p.(Arg165His)	*Missense*	Likely pathogenic	Known	Diabetes, CKD
8	c.526C > T p.(Gln176*)	*Nonsense*	Pathogenic	Known	Diabetes, CKD (kidney and pancreas tx)
9	c.526C > T p.(Gln176*)	*Nonsense*	Pathogenic	Known	Diabetes, CKD, cholestatic liver disease
10	c.493C > T p.(Arg165Cys)	*Missense*	Pathogenic	Known	Diabetes, CKD (kidney and pancreas tx)

^a^*tx* transplantation

## Discussion

The diagnosis of HNF1B-MODY is challenging in view of its heterogeneous phenotype. In this observational study, the authors describe 10 patients with several *HNF1B* molecular defects inducing different clinical presentations with a significant delay (16.5 years) between the diagnosis of diabetes and that of HNF1B-MODY.

All the patients had diabetes. The clinical presentation of diabetes did not stay in line with the usual milder forms of the disease, typical for other types of MODY, as previously described [17]. There are some subtypes of MODY that have little or no insulin therapy requirement for years. However, in HNF1B-MODY there is a combination of insulin deficiency and hepatic insulin resistance, explaining why they often require insulin therapy in the first years after diabetes diagnosis [17]. The median age at diabetes diagnosis was 28 (IQR 24) years and 60% of patients needed insulin at diagnosis which is in line with literature [14]. In this study diabetic ketoacidosis occurred in 2 patients. MODY is characterized by the absence of islet autoantibodies and by detectable serum C-peptide. Interestingly, patient 7 presented not only with diabetic ketoacidosis at 9 years-old but also with anti-GAD autoantibodies. The antibody titer was described as positive at diabetes diagnosis, but the exact value was not available in clinical files. The patient had an initial diagnosis of type 1 diabetes. After almost 20 years, the presence of CKD before diabetes onset and the presence of detectable C-peptide (0.9 ng/mL) led to the suspicion of another type of diabetes. On last follow-up anti-GAD titer was 1.4 U/mL (negative < 1.0 U/mL). In this context one could suspect a hybrid diabetes (type 1 diabetes and HNF1B-MODY). However the detectable C-peptide after 20 years of diabetes duration would exclude the possibility of a type 1 diabetes. Moreover, although the absence of the pancreatic anti-GAD and/or IA2 has been shown to discriminate Type 1 diabetes from MODY with a sensitivity of 99% and specificity of 84%, positive pancreatic antibodies have been reported in few patients (< 1%) with MODY [19, 20]. Only one patient was medicated with oral agents. This patient has the shorter duration of diabetes. She was first diagnosed with gestational diabetes in the first trimester of pregnancy. The history of CKD in childhood and the presence of bicornuate uterus led to a suspicion of HNF1B-MODY that was confirmed by genetic testing. On last observation she was medicated with metformin 2 g/day and gliclazide 30 mg/day with good glycemic control. Regarding the patients treated with insulin, 6 patients started insulin at diabetes onset, 2 patients started insulin 5 years after diabetes diagnosis and one patient 25 years after. All these patients follow a regimen of multiple daily injections of basal/bolus insulin. The mean 22.1-years diabetes duration in these patients probably explains the need for multiple daily injections scheme compared with the only patient medicated with oral agents with a 2-years diabetes duration. No other differences were found between the patients treated with insulin vs. oral agents at diabetes diagnosis namely the presence of CKD, renal malformations, or elevated liver enzymes.

Five patients had transplantation. The management of post-transplant diabetes was different among the patients depending on the presence or not of a previous diabetes diagnosis and the kind of transplantation. The patients submitted to a sole kidney transplantation and kidney/liver transplantation were managed with neutral protamine Hagedorn (NPH) insulin given in the morning. Prandial fast acting insulin was added when needed. The patient submitted to pancreas and kidney transplantation that had early pancreas graft loss maintained intensive insulin therapy with higher needs of total daily dose of insulin secondary to corticosteroids and immunosuppressants. The other patient needed a total daily dosage of insulin NPH between 10 and 14 UI/day in the first 2 weeks. This hyperglycemia after transplantation was initially interpreted secondary to methylprednisolone pulses according to the transplantation protocol. However during follow-up there was a progressive C-peptide reduction and the need of higher doses of insulin. A pancreatic graft failure was admitted.

Long-term diabetes complications were present in the patients with the longest diabetes duration (median 21 years) with a median HbA1c 9.3%. As reported in literature, the rate of cardiovascular and microvascular complications among the patients with HNF1B-MODY is similar to that of patients with type 1 and type 2 diabetes (15). In our study the longer diabetes duration and poorer control were associated with the long-term complications.

Kidney disease is a major feature of HNF1B-MODY. Most patients in this sample had the diagnosis of CKD before the diagnosis of diabetes or shortly after. This is a critical aspect to consider the diagnosis of HNF1B-MODY. Kidney malformations were present in half of the patients, which is in agreement with the literature [3, 7, 14] and unilateral kidney hypoplasia was the dominant kidney morphological abnormality. HNF1B-MODY has often been referred to as Renal Cysts and Diabetes (RCAD) syndrome [21], with a prevalence of cysts in 75% of patients in a previous study [22]. The renal ultrasound reports before transplantation were not available. In the patients without renal transplantation, kidney cysts were found in 2 (2/5). The finding of renal cysts in this sample was less frequent than expected, but it could be explained by the lack of information from transplant patients. The high prevalence (50%) of kidney transplantation observed in this series confirms the poor renal prognosis of HNF1B-MODY patients.

Regarding extra-pancreatic manifestations, the number of patients that presented with elevated liver enzymes (40%), hyperuricemia (50%) and hypomagnesemia (10%) is smaller than what is described in literature and other series [7, 22]. At last follow-up high blood pressure was present in 9 patients (9/10). All the patients with renal malformation had a diagnosis of hypertension in childhood. After renal transplantation one patient left the antihypertensive drugs with well controlled blood pressure (patient 5). All the other patients developed high blood pressure later in life. The authors did not find any association between the development of high blood pressure and genetic variant. Patient 8 and 9 (*HNF1B* variant c.526C > T p.(Gln176*)) developed hypertension in childhood and adulthood, respectively. The main difference was the presence of renal malformation in patient 8 and this seems to be the main factor for the development of arterial hypertension at a young age.

As intellectual disability has been reported in up to 10% of patients, it would be interesting to explore this point in this study. However no patient had a complete neurocognitive assessment during follow-up. The authors can only conclude about the patient’s usual medication—antidepressant drugs (70%), anxiolytic (50%) and antipsychotic (30%).

Additionally, the authors did not find any correlation between phenotype and genotype, in contrast with what previously described in larger series patients with *HNF1B* molecular alterations in which a more severe kidney disease was observed in patients with pathogenic variants compared to patients with whole gene deletion [22]. The clinical picture of the same variant varies significantly between patients, even within the same family. Patient 3 and 4, daughter and mother respectively, have distinct phenotypes despite having the same genotype. Patient 3 presented with CKD in childhood and diabetes 10 years later and presented also with altered liver tests; patient 4 (her mother) had the diagnosis of diabetes by the age of 42 with no CKD or liver disease. Diagnosis of type 2 diabetes was assumed until genetic study revealed that she carried the same *HNF1B* variant than her daughter. The same event was observed in patients 7 and 8: patient 7 (son) presented with CKD, renal malformations and diabetes in childhood while his mother (patient 8) had the diagnosis of diabetes at 40 years old and developed CKD 19 years later, probably as a diabetes long-term complication. The reason for this variability in the phenotype is not completely understood. It has been suggested that microenvironment modifiers, stochastic variation in temporal *HNF1B* gene expression and other genes may influence this phenotypic diversity [22].

To the best of our knowledge, this is the first study on HNF1B-MODY from a nationally representative cohort of patients. The main strength of this research is the evidence that the same variant could be associated with different phenotypes which is not frequently described in literature. Two novel variants were reported—a likely-pathogenic variant (patient 5) and a variant of unknown significance (patient 6). This last variant identified in heterozygosity was found in the population database gnomAD with an allele frequency of 1:251188 in the general population. Predictive computational methods (Polyphen-2 and MutationTaster) are in agreement regarding the likely functional impact of this variant and therefore it was designated as a variant of uncertain significance [23]. However it requires further testing of the family members for a better understanding of the potential pathogenicity. Other limitations of this research include the relatively small sample size compared with other similar studies. This study was based on a single-center observational cohort and lacked a uniform prospective intervention; not all clinical features were systematically assessed which limits our conclusions. Given this limitation, a protocol will be implemented in our center regarding the approach to extra-pancreatic manifestations in these patients. The present findings should be further explored in a prospective study.

## Conclusion

To the best of our knowledge, this is the largest national series of patients with HNF1B-MODY at a single center. Despite being a rare disease, HNF1B-MODY is underdiagnosed and often classified as another type of diabetes. It should be considered in patients with diabetes and CKD especially when occurring at a young age, if family history is suggestive and nephropathy appears before/shortly after the diagnosis of diabetes. Presence of liver disease and genitourinary malformations increase the degree of suspicion. Early diagnosis allows timely interventions to minimize the risk of complications and it is critical to carry out familial genetic screening and pre-conception genetic counseling. A coordinated care plan should be implemented for these patients.

## Data Availability

The datasets used and/or analysed during the current study are available from the corresponding author on reasonable request.

## Abbreviations

BMI: Body mass index
BRH: Bilateral kidney hypoplasia
CKD: Chronic kidney disease
DN: Diabetic neuropathy
DR: Diabetic retinopathy
eGFR: Estimated glomerular filtration rate
ICM: Ischemic cardiomyopathy
IQRS: Inter-quartile ranges
*HNF1**B*: Hepatocyte nuclear factor 1β
MODY: Maturity-onset diabetes of the young
SK: Solitary kidney
Tx: Transplantation
URH: Unilateral renal hypoplasia
